# Holistic Nursing Upon the Knowledge on Care During Myelosuppression among Cancer Patients

**DOI:** 10.31557/APJCP.2020.21.4.1089

**Published:** 2020-04

**Authors:** Lizy Sonia Benjamin

**Affiliations:** *Female College of Nursing, King Khalid University, Abha, Saudi Arabia. *

**Keywords:** Holistic nursing, Myelosuppression, compliance to therapeutic regimen

## Abstract

**Background:**

A study was conducted to assess the effectiveness of holistic nursing intervention upon the knowledge regarding care during myelosupression among patients with cancer at a selected hospital in Chennai, India.

**Methods:**

A quantitative research approach of quasi experimental non-equivalent with control group before –after design (non randomized) was used. The investigator included 204 participants by using purposive sampling technique which included 102 each in study and comparison group. Pre-test was done before the intervention of holistic nursing to both comparison and study group participants. Holistic nursing intervention was implemented for study group whereas comparison group received routine care. Post test was done by using the structured questionnaire after 1month. The responses from the participants were coded and statistically analyzed by using descriptive and inferential statistics.

**Results:**

The knowledge scores obtained by study group was significantly higher (13.32+2.94) when compared to comparison group (8.12+2.04). There was a statistically significant difference between study and comparison group participants, at p< 0.001. With regard to the dimensions of knowledge related to disease condition and signs and symptoms were higher in the study group when compared to the comparison group. The difference was statistically significant at p<0.001. With regard to the management and prevention, the scores were almost similar in both the groups.

**Conclusion:**

The researcher concludes with the fact that holistic nursing is a non invasive intervention, which allows cancer patients to relax, improves compliance to therapeutic regimen during myelosuppression.

## Introduction

The global burden of cancer is increasing, hitting hardest in the developing countries that have the fewest resources to fight the disease. Cancer is the second leading cause of deaths globally and is responsible for an estimated 9.6 million deaths in 2018 (WHO, 2018). Globally about 1 in 6 deaths is due to cancer.1 The numbers of new cancer cases and cancer deaths were extracted from the GLOBOCAN 2018 database that in the developed countries, cancer is the second leading cause of death accounting for 21% with 2.5 million of all mortality (Bray et al., 2018). 

Chemotherapy is the standard remedy for patients with cancer. Myelosuppression is a common and expected side effect of novel therapies for cancers. Consequences include potentially life-threatening febrile neutropenic episodes, intravenous antibiotic treatment and prolonged hospitalization. It has been reported that chemotherapy dose reductions and delays are common sequelae and may affect treatment outcomes adversely (Doshi et al., 2012). Myelosuppression refers to a decrease in the production of blood cells. Myelo is a Latin term referring to marrow. As a result of myelosuppression, patients may experience anemia, neutropenia and thrombocytopenia. Death occurring after chemotherapy usually results either from infection related to drug induced leucopoenia or from bleeding related to thrombocytopenia.

All chemotherapy patients and most radiation patients experience some form of immunosuppressant for a short duration. Depending on their severity, these adverse effects can impact patients’ medical treatment and quality of life by interrupting or reducing therapy and causing life threatening complications. These side effects variously affect the patient in physical, psychological, social and spiritual level, thus minimizing the quality of life. The more symptoms or more distress due to the symptoms experienced by patients, the lower they rate their quality of life. This correlation of the experience of symptoms and quality of life receives increasing attention in both research and clinical practice by professionals in recent years (Kirkova, 2006).

Physiologically they can lead to life threatening complications and therapy interruption. Psychologically patients can experience a limited ability to interact or work outside the home, resulting in depression, anger, financial burden and a sense of social isolation.

The American Nursing Association defined holistic nursing as all nursing practices that have healing the whole person as its goal (Hess, 2011). A holistic nurse recognizes and integrates the principles and modalities of holistic healing into daily life and clinical practice. It encourages nurses to integrate self-care, self responsibility, spirituality and reflection in their lives. 

In a descriptive cross sectional study conducted (Dhashan et al., 2015) among 100 nurses working in critical care unit at Menoufiya University Hospital, Egypt aimed at exploring and describing nurses’ perception of the meaning of holistic nursing care. The findings of this study revealed that majority (89.7%) of nurses in critical care units had high perception regarding holistic nursing care and nearly about three fourth (73.8%) of them had high perception in wards. On the other hand, the lowest percentage (10.3%) of studied nurses in critical care units had poor perception and about one fourth (26.2%) of them in wards had poor perception. This means that the majority of nurses were aware of its significance on patient outcomes.

The present study was a felt need of the researcher who was interested to address physical issues in myelosuppressed patients, as in India very few studies had been conducted and there exists a gap of knowledge. Thus, exploring in the area of holistic nursing as an intervention protocol will improve knowledge and compliance to therapeutic regimen.

This present study aims to evaluate the effectiveness of holistic nursing upon the knowledge regarding care during myelosuppression among patients with cancer at a selected hospital in Chennai.

## Materials and Methods

A quantitative research approach of quasi experimental-before–after design with a comparison group (non-randomized) was used to find out the effectiveness of holistic nursing intervention upon the knowledge regarding care on myelosuppression among cancer patients admitted at a selected tertiary hospital in Chennai. 

The objective of the study was to assess and evaluate the effect of holistic nursing upon the knowledge on care during myelosuppression among cancer patients. Holistic nursing comprising of planned teaching program and counselling on physical aspects of care related to anemia, thrombocytopenia and neutropenia, medication, nutrition, prevention of injury, prevention of infection through lecture and handouts followed by music therapy comprising of pre recorded instrumental music was administered. Institutional Ethical committee approval was obtained from the setting of data of collection.

H01- There will be no significant difference in the level of knowledge regarding care during myelosuppression before and after holistic nursing among cancer patients.

*Sample size determination*

In this study sample size was estimated based on the results of previous study of newly diagnosed patients with head and neck cancer. Thus the sample size was arrived 102 in study and comparison groups. 

*Criteria for sample selection*

*Inclusion criteria*

Male or female patients above 21 years of age, recently diagnosed to have hematological, head and neck and breast cancer of less than one year duration, from stage I to stage III on chemotherapy or radiation therapy, having anyone of parameters of Myelosuppression of low absolute neutrophil count (ANC) of less than 2,000/ cu mm, platelet less than 1,00,000/ cu.mm, hemoglobin less than 12 gm% and able to read and understand Tamil and or English were included for the study.

*Exclusion criteria*

The subjects who were disoriented and confused, cognitive impairment, Stage IV, hemodynamically unstable and terminally ill patients were excluded.

The study was conducted at a selected tertiary care oncology hospital at Chennai and Ethical committee permission was obtained by the investigator from the Institutional Ethical committee. The investigator recruited 102 participants each for study and comparison group using non-probability purposive sampling technique based on the inclusion criteria.

A structured instrument was used to collect the data as discussed below.

*Part A: Demographic Variable Profile*

It includes items such as the age, gender, marital status, occupation, education and place of residence of myelosuppressed patients which were obtained by structured interview schedule.

*Part B: Clinical variable Profile*

It includes items like diagnosis, duration of illness, staging of malignancy, primary treatment, family history of cancer, co-morbidities present, hemoglobin, WBC count and platelet count obtained through clinical records survey.

*Part C: Structured Knowledge Questionnaire*

This questionnaire was prepared by the investigator used for assessing the knowledge related to care during myelosuppression among patients with cancer. There are 25 items with responses graded on a 4 point scale. The maximum score is 25 and the minimum score is 0. The questions were classified based on 4 dimensions that include disease condition, signs and symptoms, management and prevention of complications. The reliability of the tool was tested using test retest reliability of 0.85.

*Scoring interpretation*

Obtained score is interpreted as follows: 

75-100          Adequate Knowledge

50 – 74          Moderately Adequate Knowledge

<50          Poor Knowledge

Thus the total obtainable score is 0 -100.

*Data collection*

The study was conducted after obtaining clearance from Ethical committee, Apollo Hospitals, Chennai. Consent was obtained from all the participants, bystander before the data collection. Confidentiality was maintained throughout the study.

The demographic data were collected by interview with the patients. Pretest was conducted using a structured knowledge questionnaire on care during myelosuppression by using the predetermined tools. After the pretest, holistic nursing comprising of planned teaching programme related to care of anemia, thrombocytopenia and neutropenia, medication, nutrition, prevention of injury, prevention of infection followed by music therapy comprising of prerecorded instrumental music was administered on the same day through headphone for a period of one hour, once in the morning and once in the evening for 10 days continuously. The types of instrumental music were sitar, violin, piano, strings, and flute. This intervention was given on one to one basis.

The participants were selected through purposive sampling technique and selected samples were assigned to study and comparison groups (102, 102). The subjects in the study group received holistic nursing intervention. A pre test and post test assessment after 1 month duration was done for the subjects in the study and comparison groups were compared before and after the intervention to test the effectiveness of the nursing intervention. The instruments used for the study consisted of proforma to assess demographic and clinical variable profile, structured questionnaire to assess the knowledge on the care during myelosuppression.

Descriptive and inferential statistics were used to analyze the data by using SPSS version 22.0. 

## Results

Table 1 illustrates that a baseline comparison of the demographic characteristics was done between both the groups to ascertain homogeneity. The present study revealed that in the study group, the majority of the study participants were between the age group of 31-60 years, 71.56 %, females 58.82%, belongs to nuclear family 66.66%, with graduate level of education 55.88%, engaged in sedentary work 48.03%, earning monthly income of more than Rs50,000/- 66.66%, non vegetarian 76.47%, and 66.66% residing in urban area. 

In the comparison group, 70.58% belongs to the age group of 31- 60 years, females 61.76%, 70.58% belongs to nuclear family with graduate level of education 51.96%, engaged in sedentary work 49.01%, earning monthly income of more than Rs50,000/- 69.60%, non vegetarian 74.50%, and 56.86% residing in urban area.

Table 2 denotes the clinical variables in the study group, on admission majority 64.70%, of the study participants were diagnosed to have hematologic malignancy since more than 3 months 53.92% and 50% were found to be in Stage I with primary treatment as chemotherapy 50.98% no family history of Cancer with 68.62%, Hemoglobin 10-12gm% 45.09%, neutrophil less than 1,500/ cu.mm 58.82% and platelet count less than 75,000/cu.mm 45.09%.

In the comparison group of clinical variables, on admission majority 63.72% of the patients were diagnosed to have hematologic malignancy since more than 3 months 52.94% and 45.09% were found to be in Stage I with primary treatment as chemotherapy 58.86%, no family history of Cancer with 70.58%, Hemoglobin 10-12gm% 50%, neutrophil lower less than 1,500/ cu.mm 56.86% and 47.05% with platelet count less than 75,000/cu.mm.

A baseline comparison of the demographic and clinical characteristics was done between the study and comparison groups to ascertain homogeneity and thus both the groups were homogenous.

**Figure 1:**
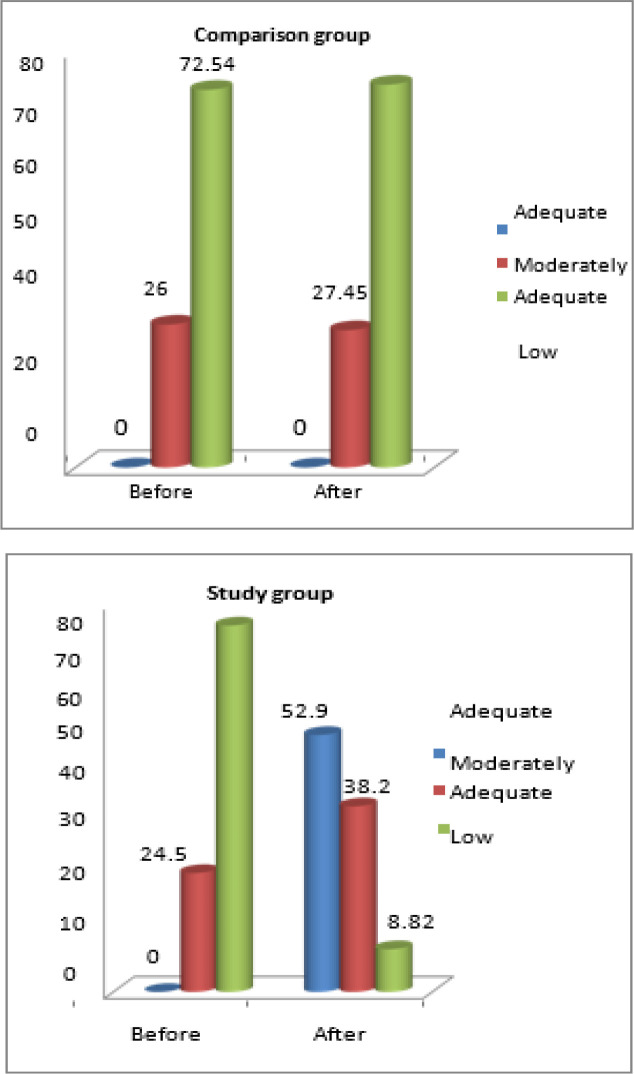
Percentage Distribution of Level of Knowledge Scores before and after Holistic Nursing in Patients with Cancer

Figure 1 highlights majority of the cancer patients 52.94% had adequate knowledge 38.23% had moderately adequate knowledge and 8.82% had low level of knowledge in the study group whereas in the comparison group 27.45% had moderately adequate knowledge and 72.54% had low level of knowledge after holistic nursing intervention.

From Table 3 it is evident that when independent t test was used to compare the post test scores of knowledge between study group (13.32+2.94) and comparison group (8.12+2.04) it denoted that there was a statistically significant difference between study and comparison group participants, at p< 0.001. It was noticeable from these findings that the knowledge scores were better among study than in comparison group. This shows the effectiveness of holistic nursing upon knowledge scores. Hence H01 there will be no significant difference in the level of knowledge regarding care on myelosuppression before and after holistic nursing among patients with cancer was rejected.

Table 4 indicates that when paired t test was used to compare the prettest and posttest scores with regard to the dimensions of knowledge related to disease condition and signs and symptoms were higher in the study group when compared to the comparison group. The difference was statistically significant at p<0.001. With regard to the management and prevention, the scores were almost similar in both the groups as neutropenic precautions were followed in both the groups since they are more susceptible to infection, patients with neutropenia take extra precautions, such as avoiding crowds, maintaining a low microbial diet and routinely monitoring for breaks in the skin or oral mucositis. Hence the hypothesis H01 “there will be no significant difference in the level of knowledge regarding care on myelosuppression before and after holistic nursing among patients with cancer” was rejected.

**Table 1:** Distribution of Patients According to Demographic Characteristics (N=204)

Demographic Variables	Comparison Group (n=102)		Study Group (n=102)		p value
	f	%	f	%	
Age in years					
< 30	25	24.50	22	21.56	
31-60	72	70.58	73	71.56	0.42
>60	5	4.90	7	6.86	
Gender					
Male	39	38.23	42	41.17	0.66
Female	63	61.76	60	58.82	
Educational status					
Illiterate	17	16.66	16	15.68	
Higher secondary	32	31.37	29	28.43	0.03
Graduate and above	53	51.96	57	55.88	
Marital status	27	26.47	28	27.45	
Single					
Married	58	56.86	59	57.84	0.03
Widowed	17	16.66	15	14.70	
Current occupation					
Employed	64	62.07	69	67.64	
Unemployed	18	17.64	7	6.86	
Retired	10	9.80	21	20.58 4.67	
House wife	10	9.80	5	4.90	0.86
Income					
10,000 – 20,000	6	5.88	8	7.84	
20,000 – 50,000	25	24.5	26	25.49	
> 50,000	71	69.60	68	66.66	0.77
Nature of work					
Sedentary work	50	49.01	49	48.03	
Moderate work	39	38.23	38	37.25	
Heavy work	13	12.74	15	14.70	0.71
Dietary pattern					
Vegetarian	26	25.49	24	23.52	0.54
Non vegetarian	76	74.50	78	76.47	
Area of residence					
Rural	30	2.94	34	33.33	
Urban	72	70.58	68	66.66	0.21
Type of family					
Joint	30	2.94	34	33.33	0.21
Nuclear	72	70.58	68	66.66	

NS, Not significant

**Table 2:** Distribution of Patients According to Clinical Characteristics (N=204)

Clinical Variables	Comparison Group (n=102)		Study Group (n=102)		p
	f	%	f	%	value
Duration of present illness					
< 1 month	18	17.64	17	16.66	0.11
1- 3 months	30	29.41	30	29.41	
> 3 months	54	52.94	55	53.92	
Staging for Malignancy					
Stage I	46	45.09	46	45.09	0.18
Stage II	36	35.29	28	27.45	
Stage III	20	19.60	28	27.45	
Stage IV	-	-	-	-	
Diagnosis					
Hematological Malignancies	65	63.72	66	64.70	0.41
Head and neck cancers	29	28.43	30	29.41	
Breast cancer	8	7.84	6	5.88	
Primary treatment					
Chemotherapy	58	58.86	52	50.98	0.34
Radiation therapy	36	35.29	38	37.25	
Surgery	8	7.84	10	9.80	
Presence of co-morbid illness					
Present	52	50.98	54	52.94	0.47
Not present	50	49.01	48	47.05	
Family history of cancer					
Present	30	29.41	32	31.37	0.14
Not present	72	70.58	70	68.62	
Hemoglobin level (g%)					
10-12	46	45.09	51	50	0.14
< 10.0–8.0	40	39.21	38	37.25	
< 8.0–6.5	16	15.68	13	12.74	
< 6.5	-	-	-	-	
Neutrophil count					
< LLN – 1,500	60	58.82	58	56.86	0.48
< 1,500 –1,000	28	27.45	31	30.39	
< 1,000– 500	14	13.72	13	2.74	
< 500	-	-	-	-	
Platelet count	48	47.05	48	47.05	
< LLN -75,000					0.21
< 75,00 –50,000	40	39.21	41	40.19	
<50,00 –25,000	14	13.72	13	2.74	

**Table 3:** Comparison of Mean of Knowledge Scores before and After Holistic Nursing among Patients with Cancer (N=204)

Groups (n=102)	Pretest		Independent	Posttest		Independent
	Mean	SD	t test	Mean	SD	t test
Comparison group	4.26	1.31	8.12 NS	8.52	2.04	13.32***
Study group	4.12	1.76		13.26	2.94	

NS, Not significant, *** p<0.001

**Table 4:** Comparison of Mean on Dimensions of Knowledge Scores before and After Holistic Nursing among Patients with Cancer

Dimensions	Comparison group		p value	Study group		p value
	Mean	SD		Mean	SD	
Disease condition (0-4)			p=1.00 NS			p=0.001***
Pretest	0.74	0.68		1.02	0.77	
Posttest	0.74	0.71		2.55	0.49	
Signs and symptoms (0-5)			p=0.80 NS			p=0.001***
Pretest	1.34	0.60		1.30	0.65	
Posttest	1.36	0.57		3.78	0.62	
Management (0-6)			p=0.001***			p=0.001***
Pretest	1.51	0.50		1.49	0.50	
Posttest	2.00	0.00		4.50	0.50	
Prevention			p=0.001***			p=0.001***
Pretest	1.26	0.44		1.36	0.48	
Posttest	1.45	0.50		9.26	0.44	

NS, Not significant, ***p<0.001

## Discussion

*Demographic and clinical characteristics*

The present study revealed that most of the participants (71.56%, 70.58%) were aged 31 – 60 years in the study and comparison group. Thus the participants in this study too constituted of mostly young subjects. Findings from a similar study attributed that the median age of experiencing neutropenia was 49 years and 56 years conducted by (Doshi et al., 2012).

In the present study, on admission (58.82%, 56.86%) had neutrophil lower less than 1,500/ cu.mm in the study and comparison group. The above result was supported by a similar study that revealed 65% of the hospitalizations for febrile neutropenia occurred in the first 2 cycles of chemotherapy for intermediate grade Non-Hodgkin’s Lymphoma (Morrison et al., 2001). In addition, in patients with advanced breast carcinoma 75% of the episodes of Febrile Neutropenia occurred during the first cycle course of chemotherapy (Cameron D, 2001).

In the present study, on admission (50%, 45.09%) of patients had Hemoglobin 10-12 gm%, neutrophil lower less than 1,500/ cu.mm (56.86%, 58.82%) and platelet count less than 75,000/cu.mm (47.05%, 45.09%) in the study and comparison group respectively. The findings are congruent with similar study reported to have anemia with hemoglobin ≤ 12 g/dl is present in 73% of patients at initial diagnosis of multiple myeloma. It can be seen in 97% at some time during the course of the disease. Leukopenia with white blood cell count ≤ 4 x 109/L is seen in 20% of patients at time of diagnosis. Thrombocytopenia with platelet count < 100 x 109/L is seen in 5% of patients at time of diagnosis (Birgegård G, 2006). 

Further the univariate analysis demonstrated no significant difference between study and comparison groups. 

When paired ‘t’ test was used to compare the pre and post test scores of knowledge between study and comparison group, it denoted that there was a statistically significant difference between study and comparison group participants, at p< 0.001. The study findings revealed that the overall knowledge scores obtained by study group was significantly higher (M=20.58) at (p< 0.001) after holistic nursing when compared to comparison group (M= 4.85). It was noticeable from these findings that the knowledge scores were better among the study than in comparison group. 

The results can be attributed by a similar study conducted by (Lavdaniti, 2017). In a survey conducted among 166 cancer patients and 95 nurses conveniently recruited from three major hospitals in Adelaide, Australia to identify the level of agreement between cancer patients and nurses about cancer patients’ QoL. Each patient and nurse was invited to complete the World Health Organization Quality of Life Brief (WHO QoL-BREF) questionnaire separately. This questionnaire considers QoL across four domains or dimensions: physical health, psychological health, social relationship and environment. The results highlighted that the proportion of the exact agreement between the two groups was 34.9%, 34.5%, 33.8% and 36.9% for the physical, psychological, social relationship, and environmental QoL domains, respectively. Results indicate that nurses do not have a holistic understanding of cancer patients’ QoL. The nurses’ mean clinical experience with cancer patients was 8.15 years with a range of 0-22 years. The patients had a range of cancer diagnoses with breast cancer being the most prevalent. Most of the patients were being treated as inpatients with chemotherapy being their primary treatment. (Bahrami, 2010). 

Majority of the cancer patients 52.94% in the study group had adequate knowledge 38.23% had moderately adequate knowledge and 8.82% had low knowledge whereas in the comparison group 27.45% had moderately adequate knowledge and 72.54% had low level of knowledge with the routine nursing intervention. Similar findings are consistent with a cross sectional study conducted by (Dane et al., 2011) on cancer related knowledge among cancer patients and general public in Turkey. The results revealed that from 1.7% to 88.75% possess some false information and suggested additional training in Universities as well as continuing educational programs is recommended to inform the Turkish public about cancer. 

Another similar qualitative study (Farooqui M et al., 2011) was conducted to explore the nursing role in education and follow up of patients who were taking oral chemotherapy (CT) and to identify the worldwide gap in patient education about oral CT. 1115 oncology nurses from 15 countries were included. Findings showed that 52% had some type of guidelines/protocols, 47% reported not having received any education about oral CT drugs. While 64% report being involved in patient education, 58% of subjects indicated lack of patient education materials that are specific for oral CT agents. Only 27% stated that they gave all necessary information such as when and how to take the drugs, drug safety and storage, side effects, and symptom management. Findings revealed the need for professional education for nurses to ensure comprehensive, consistent patient education and development of written materials for patients receiving oral CT treatment (Kav et al., 2008).

This study highlighted there was also significant difference on all the dimensions of knowledge. Thus holistic nursing was an effective nursing intervention. The study group was higher compared to comparison group reflects the effectiveness of holistic nursing. Thus providing holistic care is a component of providing integrative care. Integrative care utilizes conventional and complementary approaches to cancer integrating them with a focus on the whole person, which includes emotional, spiritual, social, and lifestyle (diet, physical activity, sleep, relationships) factors, the provider-patient relationship as a partnership and interprofessional collaboration. (Cadet et al., 2016). 

Music therapy is an effective form of supporting cancer care for patients during the treatment process. It may be also basic for planning effective programs of rehabilitation to promote wellness, improve physical and emotional well-being and the quality of life. While some types of cancer are best addressed with a single type of treatment, others are treated through a combination of surgery, chemotherapy and/or radiation therapy. The music therapy program is applied to meet patients’ needs during diagnosis and treatment and is practiced with both individual patients and patient groups. Music therapy is mainly used to promote relaxation, reduce anxiety and stress; relieve discomfort; reduce patients’ experience of pain; and offset some of treatment related symptoms. Music therapy offers opportunities for self-expression and brings positive experiences. (Stanczyk, 2011).

The study findings revealed that there was a highly significant difference between the study and comparison group on overall mean knowledge scores and on all the dimensions of knowledge. The mean scores of overall knowledge obtained by study group and comparison group was significantly (p< 0.001) higher in study group (M=20.58) after holistic nursing when compared to comparison group (M= 4.85).

Similar findings have been reported by (Devine et al., 1995) in a meta analysis intervention studies found that patients with cancer receiving psycho educational or psychosocial interventions showed much lower rates of anxiety, depression, mood disorders, nausea, vomiting and pain and significantly greater knowledge about disease and treatment, than the comparison group.

Music therapy is a growing discipline and includes diverse practices and models used worldwide. In developed countries, music therapy in cancer care is an emerging field. Music therapy is non invasive and free of side effects, it is being integrated into the standard care in major cancer hospitals to help relieve pain and other physical and psychological discomfort (Boso, 2006).

It is validated from the above that holistic nursing intervention was effective to promote the knowledge on self care management among myelosuppressed patients. Hence, “H01–There will be no significant difference in knowledge regarding care on myelosuppression among patients with cancer” was rejected.

Thus educating patients on their condition can go a long way in managing myelosuppression. Nursing efforts should focus upon educating the patient and their family, based on the treatment recommendations.

*Implications for nursing practice*

Oncology nurses are uniquely positioned to step into new roles emphasizing patient and family education and support. The concept of holistic nursing care must be emphasized in the undergraduate as well as the postgraduate curricula to prepare candidates to apply the established programs where they work. Short term courses on holistic nursing and its application can be conducted in nursing educational institutions. The primary goal of training programs is to produce competent practitioners. Thus nurses need to develop competencies to work in myelosuppressed unit.

In conclusion, the researcher concludes with the fact that holistic nursing is a non invasive intervention, which allows cancer patients to relax, gains knowledge and are compliant to therapeutic regimen during myelosuppression. Hence oncology nurses need to come in frontline with clinical investigations emphasizing to assess the particular needs in cancer patients during myelosuppression as well as to test effect of nursing interventions in various domains of care on clinical outcome. Nursing assessment and education of patients play a crucial role too. Nurses can independently practice holistic nursing and can implement such effective practices for patients.
